# How Task Constraints Influence the Gaze and Motor Behaviours of Elite-Level Gymnasts

**DOI:** 10.3390/ijerph18136941

**Published:** 2021-06-29

**Authors:** Joana Barreto, Filipe Casanova, César Peixoto, Bradley Fawver, Andrew Mark Williams

**Affiliations:** 1 Universidade de Lisboa, Faculdade de Motricidade Humana, Laboratório de Perícia no Desporto, CIPER, Cruz Quebrada Dafundo, 1495-751 Lisboa, Portugal; 2 Faculty of Physical Education and Sport, University Lusófona of Humanities and Technology, 1749-024 Lisbon, Portugal; p5661@ulusofona.pt; 3 Center of Research, Education, Innovation and Intervention in Sport (CIFI2D), Faculty of Sport, University of Porto, 4200-450 Porto, Portugal; 4 Universidade de Lisboa, Faculdade de Motricidade Humana, Laboratório de Perícia no Desporto, Cruz Quebrada Dafundo, 1495-751 Lisboa, Portugal; cpeixoto@fmh.ulisboa.pt; 5 US Army Medical Research Directorate-West, Walter Reed Army Institute of Research, Joint Base Lewis McChord, Pierce, WA 98433, USA; bradley.j.fawver.ctr@mail.mil; 6 Department of Health and Kinesiology, College of Health, University of Utah, Salt Lake City, UT 84112, USA; Mark.Williams@health.utah.edu

**Keywords:** expertise, inertial sensors, kinematic analysis, perception-action coupling, performance, visual fixation

## Abstract

Perception-action coupling is fundamental to effective motor behaviour in complex sports such as gymnastics. We examined the gaze and motor behaviours of 10 international level gymnasts when performing two skills on the mini-trampoline that matched the performance demands of elite competition. The presence and absence of a vaulting table in each skill served as a task-constraint factor, while we compared super-elite and elite groups. We measured visual search behaviours and kinematic variables during the approach run phase. The presence of a vaulting table influenced gaze behaviour only in the elite gymnasts, who showed significant differences in the time spent fixating on the mini-trampoline, when compared to super-elite gymnasts. Moreover, different approach run characteristics were apparent across the two different gymnastic tasks, irrespective of the level of expertise, and take-off velocity was influenced by the skill being executed across all gymnasts. Task constraints and complexity influence gaze behaviours differed across varying levels of expertise in gymnastics, even within a sample of international level athletes. It appears that the time spent fixating their gazes on the right areas of interest during the approach run is crucial to higher-level performance and therefore higher scores in competition, particularly on the mini-trampoline with vaulting table.

## 1. Introduction

Gymnasts perform highly complex skills on diverse apparatus, each with specific characteristics and requirements [1]. Within the Teamgym discipline, athletes perform skills using only the mini-trampoline (MT) and using the mini-trampoline with vaulting table (MTVT). Both skills require continuous perception-action coupling in order to visually regulate motor behaviour, particularly during the approach run [2,3,4,5,6,7]. The mechanisms underlying performance are believed to vary by apparatus and skill complexity, notably with the inclusion of a vaulting table on MTVT skills; however, our understanding of how task constraints influence gaze and motor behaviours during the approach run to the MT and MTVT remains limited.

The need for on-line visual regulation during the approach run when vaulting has been previously confirmed [4], with the springboard and vaulting table considered important sources of visual information [2,3,7]. Gymnasts regulate the approach run based on a visual strategy using time-to-contact information [6]. The application of direct measures of gaze behaviour (e.g., eye-tracking systems) in recent work [8,9,10,11,12] has allowed consideration of the spotting hypothesis: gymnasts fixate their gaze on specific locations in their environment when performing complex techniques, but these behaviours are dependent on the experimental condition/task and performer’s level of expertise. In a recent study with elite-level gymnasts (i.e., national team members), we demonstrated that gymnasts used different visual strategies when performing on an MT alone compared to the MTVT [12]. These data collectively suggest that elite gymnasts develop a unique visual strategy to optimally select and process relevant visual information from their environment, especially when different constraints are presented (e.g., vaulting table).

Several limitations in the existing literature prompted the present study. First, the tasks used in previous work [3,4,6,7,8,13,14] were relatively simple (e.g., single back somersault on trampoline and handspring on vault) compared to the techniques typically performed at the elite level. Second, the selection of indirect measures of gaze behaviour did not allow effective inferences about gaze and motor behaviours during skill execution. Lastly, comparisons across groups of true experts are generally lacking in this field of literature.

To address these gaps, we measured gaze and motor behaviours while international level gymnasts of varying levels of expertise (i.e., super elite and elite) performed mini-trampoline skills either in the absence (i.e., MT) or presence (i.e., MTVT) of a vaulting table. This approach is novel in addressing two key gaps in the existing literature: (1) measuring perceptual and motor variables during the execution of complex skills; and (2) the recruitment of a cohort on truly elite (national team) athletes, although in regards to the latter at the cost of a reduced sample size.

The purpose of this study is to investigate if task constraints (i.e., presence and absence of vaulting table) influence gaze and motor behaviours of elite-level gymnasts. First, we expected that gaze behaviours would be influenced by task constraints independently of participants’ levels of expertise [15]. More specifically, gymnasts were expected to fixate on the most relevant environment locations during both skills: (a) greater fixation number and longer dwell times on the mini-trampoline when performing the MT skill; and (b) a significant increase in the proportion of fixation number and dwell times on the vaulting table when performing the MTVT skill [2,3]. Second, we predicted that super elite gymnasts would exhibit longer final fixations on areas of interest (AOI; either mini-trampoline or vaulting table), compared to their less-elite counterparts; and the number of fixations was expected to be positively associated with superior performance, since a faster visual search rate in more-expert gymnasts has been reported in other related tasks [16,17,18]. Third, we predicted that motor behaviour, more specifically take-off velocity, would be influenced by task constraint with all participants increasing their take-off velocity on MTVT, due to the higher complexity of the task. Finally, we expected that greater take-off velocity would be associated with better scores [19,20,21,22].

## 2. Materials and Methods

### 2.1. Sample

Altogether, 10 elite Teamgym gymnasts, all from the Teamgym national team participated in the study. Five international Teamgym judges from Portugal, Italy and the Czech Republic scored execution on MT and MTVT according to the Teamgym Code of Points [23]. Based on the scores from judges, the participants were separated into two groups: super elite (i.e., with higher scores) gymnasts (*n* = 5; mean age: 27.8 ± 3.35 years, mean experience: 13.4 ± 2.32 years); and elite (i.e., with lower scores) gymnasts (*n* = 5; mean age: 22.6 ± 2.30 years, mean experience: 8.2 ± 1.04 years). The study was conducted in accordance with the Declaration of Helsinki and approved by the Faculty Ethics Committee (1/2020; 24/01/2020). Gymnasts reported normal or corrected to normal vision and signed an informed consent prior to starting experimental procedures.

### 2.2. Tasks

**Figure 1:**
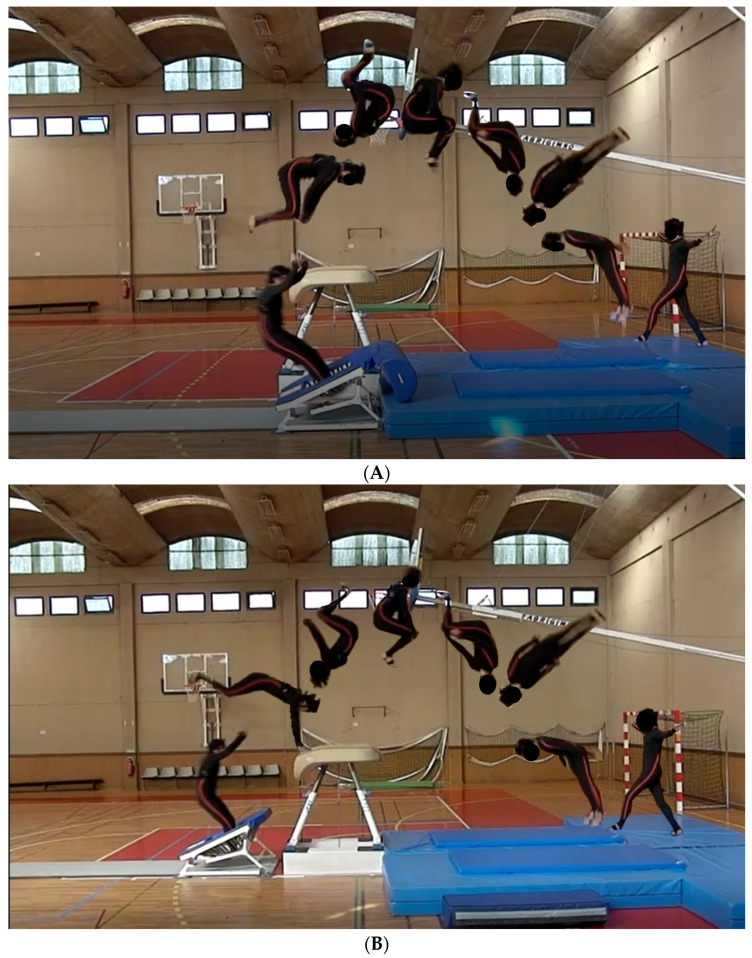
(**A**) Task performed on mini-trampoline (MT): tucked barani out. After a maximum 25 m approach run, the gymnast performs a take-off from the mini-trampoline followed by a double somersault in a tucked position (720° on transversal axis rotation), and a half twist (180° on longitudinal axis rotation) before landing. (**B**) Task performed on mini-trampoline with vaulting table (MTVT): handspring tucked barani out. After a maximum 25 m approach run, the gymnast performs a take-off from the mini-trampoline followed by a forward entrance placing their hands in vaulting table (support phase), one and a half somersault in a tucked position (total of 720° on transversal axis rotation) and a half twist (180° on longitudinal axis rotation) before landing.

Participants performed two separate skills on two apparatus: tucked barani out on MT (Figure 1A) and handspring tucked barani out on MTVT (Figure 1B). The manipulation of task constraints (i.e., apparatus) was represented by the presence (MTVT) or absence (MT) of a vaulting table. Both skills have similar movement patterns and are usually performed at elite level in this discipline.

### 2.3. Instruments and Procedure

Gaze data were sampled frame-by-frame at 50 Hz using a Tobii Pro Glasses 2 (Tobii Pro AB, Stockholm, Sweden) mobile eye-tracking system (ETS), which uses a binocular eye-movement system to measure the relative position of the pupil and corneal reflection and overlays point-of-gaze onto a video image of the scene. Tobii Glasses Controller Software running on a Dell Venue 11 Pro 7130, Windows 8/8.1 Pro tablet was used to manage eye movement recording, with images transferred to a computer and analysed using Tobii Pro Lab (Version 1.142, Tobii Pro AB, Stockholm, Sweden). The eye-movement system was calibrated by asking each participant to stand still and visually fixate on the centre of a calibration card 1.25 m away for five seconds. After calibration, participants fixated on nine different points in the environment at various distances, heights, and widths.

**Figure 2:**
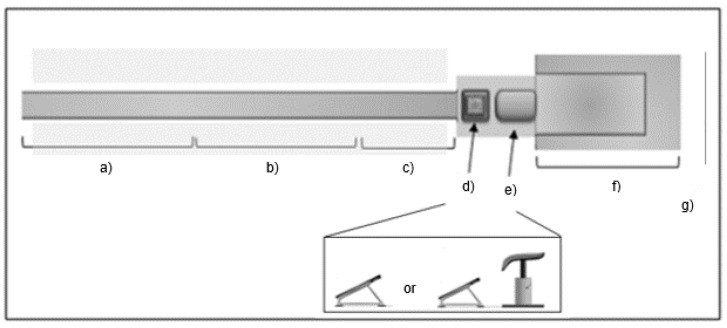
The experimental setup, apparatus, and areas of interest (AOIs) considered for analysis. AOIs included: (**a**) Start Run (10 × 2 m); (**b**) Mid Run (10 × 2 m); (**c**) End Run (5 × 2 m—grey areas); (**d**) mini-trampoline; (**e**) vaulting table; (**f**) landing mat (4 × 7 m); and (**g**) front wall. Lateral detailed view is presented at the bottom of the figure for each apparatus and AOIs: mini-trampoline without vaulting table (MT) and mini-trampoline with vaulting table (MTVT). Adapted from Hughes et al. (2013).

A visual fixation was defined as a period of at least 100 ms when the eye remained stationary within ±0.5° of movement tolerance [24]. Visual gaze behaviours were analysed to obtain search rate and dwell time data for dependent measures. Specifically, we quantified search rate from recordings of the mean number of visual fixations, the mean fixation duration in milliseconds, and the total number of fixation locations per AOI. We defined dwell time as the proportion of time spent fixating on each of eight different locations/AOIs as a percent of the total fixation time (see Figure 2). The AOIs included: (a) the first 10 m of approach run or ‘Start Run’; (b) between 10 and 20 m of approach run or ‘Mid Run’; (c) the last 5 m of approach run or ‘End Run’; (d) the floor area surrounding or ‘Floor’; (e) the mini-trampoline; (f) the vaulting table if present; (g) the landing mat; and (h) the front wall. The final fixation dwell time relative to the total duration of the approach run was calculated, as well as the final fixation duration before mini-trampoline take-off (if >100 ms).

We recorded kinematic data using a system of seventeen inertial measurement sensors (Xsens MVN Link; Xsens Technologies, Enschede, The Netherlands) at a rate of 240 Hz. The placement of sensors and calibration conformed to the manufacturer’s recommendations. Kinematic data were processed in multilevel high definition using MVN Analyse software (Version 2019.2.1, Xsens Technologies, Enschede, The Netherlands). We used acceleration peaks, representing foot impacts on the mat carpet, to define the number of steps of the approach run and contact with mini-trampoline [25]. We calculated segment positions and orientations using MVN Fusion Engine for Xsens, and these data were imported into Visual 3D (Version 6, C-Motion, Inc., Germantown, MD, USA) for dependent measure computation. We derived specific measures from the kinematic post-processing including: (a) take-off velocity in meters per second; (b) step length—mean length of the last nine steps before take-off in meters; (c) hurdle length—mean length between the last step and take-off on mini-trampoline in meters; and (d) step frequency—the number of steps per minute. Both systems were synchronized using an LED connected through a trigger signal.

Experimental tasks were performed in a sports hall according to Teamgym directives [26]. All trials were recorded using a video camera (Casio EXILIM EX-F1, 60 Hz) placed perpendicular to the MT/MTVT. Participants performed a twenty-minute warm-up and several practice trials before instrument calibration was performed.

Participants performed four trials of each skill as if they were in a competition, starting with MT, and were informed that trials would be evaluated by international Teamgym judges. Participants chose the distance of the run-up, which was marked with black tape every five meters. We repeated instrument calibrations before each trial. Following the completion of all trials, participants were fully debriefed. According to judges’ scores, we considered the best two trials performed on each apparatus for analysis (MT and MTVT) as well as computing a total score on each apparatus.

### 2.4. Statistical and Data Analysis

A between and within-participants research design was carried out. Gaze and kinematic trial durations were normalized to 100 data points to improve comparison between groups. We performed Intraclass Correlation Coefficient (ICC) and Confidence Intervals (CI) estimates for judges’ ratings. Linear mixed-effect regressions (LMERs) were utilized to examine the effects of Expertise level (Group: super elite, elite) × Task/Skill (Apparatus: MT, MTVT) on vaulting scores and whether these factors were associated with different process-tracing measures of performance (e.g., gaze behaviour, kinematics). In each model, Group and Apparatus were fixed effects, while random effects included Participant and Participant × Group. The normality of residuals was inspected using Q–Q plots, with all models exhibiting normality visually and according to the Kolmogorov–Smirnov test (all *p* < 0.05). Given the elite nature of the participants, and therefore smaller sample size available, data normality was confirmed using equivalent. We also conducted non-parametric tests of normality using the Shapiro–Wilk test. We evaluated heterogeneity using Levene’s test. In the case of deviations with respect to these tests, the measure of interest was transformed (e.g., log, mean-centred) prior to analysis.

We calculated the significance of individual parameters for each model using a *t*-test with a Welch–Satterthwaite approximation and report 95% confidence intervals for all analyses. We also report partial-eta squared (*η*^2^) effect sizes for main effects, interactions, and univariate follow-up tests, and Cohen’s *d* effect sizes are reported for pairwise comparisons. The significance level for all statistical tests was set at *α* = 0.05. Analyses were conducted using SPSS v25.0 (SPSS Inc., Chicago, IL, USA; IBM Corp, Armonk, NY, USA) or R-Studio v3.6.1.

## 3. Results

### 3.1. Performance

The ICC estimates and 95% confident intervals for judges’ scores indicated good reliability [27], with an absolute agreement value of 0.863 (CI_95%_ = 0.813, 0.904). The more-elite or super elite group (mean final score = 9.492 pts ± 0.05, out of 10) generally outperformed the less elite group (mean final score = 9.112 pts ± 0.17); however, a significant Group × Apparatus interaction was documented for score, *β*= 0.356 (0.07, 0.64), *t*(28.0) = 2.446, *p* = 0.021, *η*^2^ = 0.150. Post hoc tests revealed that when performing on MTVT, the super elite group scored significantly higher (mean score = 9.32 pts ± 0.14) compared to the elite group (mean score = 8.77 pts ± 0.37; *β* = 0.542 (0.24, 0.84), *t*(8) = 3.529, *p* = 0.007, *η*^2^ = 0.491, *d* = 1.97), but this effect was not evident on the MT (*p* = 0.126, *d* = 0.96). Scores were significantly higher for MT compared to MTVT for both the super elite (*β* = 0.320 (0.17, 0.47), *t*(18) = 4.123, *p* < 0.001, *η*^2^ = 0.486, *d* = 1.85) and elite groups (*β* = 0.676 (0.43, 0.92), *t*(14) = 5.588, *p* < 0.001, *η*^2^ = 0.656, *d* = 2.30). In summary, expertise level was positively associated with vaulting scores overall, which was driven by the more difficult constraints present during the MTVT task.

### 3.2. Gaze Behaviour

**Figure 3:**
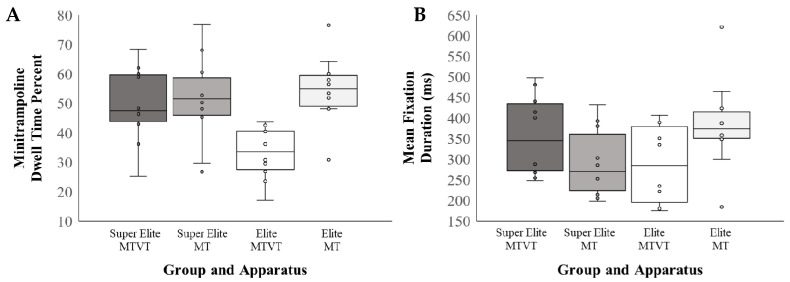
Gaze measures by group (super elite vs. elite) and apparatus (MTVT vs. MT): (**A**) dwell time percent on the mini-trampoline during the approach run, as a function of total fixation time; (**B**) mean fixation duration in milliseconds.

The mean ± SD values for search rate and gaze behaviour variables are presented as a function of apparatus in Table 1. A significant Group × Apparatus interaction was obtained for dwell time percent on the mini-trampoline, *β* = 20.040 (6.30, 33.78), *t*(28) = 8.133, *p* = 0.009, *η*^2^ = 0.205 (Figure 3A). Post hoc tests revealed that when performing the MTVT task, the elite participants spent significantly less time (~16%) fixating on the mini-trampoline (mean dwell time = 33.08% ± 8.98) compared to the super elite group (mean dwell time = 49.46% ± 13.38; *β* = 16.379 (4.61, 28.15), *t*(10) = 3.012, *p* = 0.013, *η*^2^ = 0.372, *d* = 1.46). However, there were no main effects of Group for dwell time on the mini-trampoline when executing the MT skill (*p* = 0.593, *d* = 0.27). Dwell time on the mini-trampoline was significantly greater (~21%) for the elite group when performing the MT skill compared to MTVT skill, *β* = 21.63 (13.24, 30.03), *t*(14) = 5.208, *p* < 0.001, *η*^2^ = 0.633, *d* = 2.05. In summary, participants in the elite group tended to fixate less on the mini-trampoline when performing the MTVT task (~33%), whereas dwell time on the mini-trampoline did not differ across groups for both apparatuses (~49–55%). Although the super elite group appeared to spend less time fixating the vaulting table when performing the MTVT skill (mean dwell time = 17.08% ± 6.86) when compared to the elite group (mean dwell time = 26.80% ± 10.75), this effect did not reach the threshold for significance, *β* = 9.720, *p* = 0.083, *η*^2^ = 0.228, *d* = 1.08.

A significant Group × Apparatus interaction was documented for mean fixation duration, *β =* 162.70 (48.07, 277.34), *t*(28) = 2.776, *p* = 0.010, *η*^2^ = 0.154 (Figure 3B). Post hoc tests revealed that this effect was driven by participants in the elite group demonstrating average fixation durations that were approximately 100 ms longer when performing the MT (mean fixation time = 383.21 ms ± 112.20) compared to MTVT (mean fixation time = 291.47 ms ± 83.85; *β* = 96.40 (19.96, 172.83), *t*(14) = 2.574, *p* = 0.022, *η*^2^ = 0.304, *d* = 0.923). Finally, there were no significant main effects of Group or Apparatus, or interaction effects, for the total number of fixations (*p* > 0.085), dwell time on the Mid Run (*p* > 0.676), dwell time on the End Run (*p* > 0.693), final fixation dwell time (*p* > 0.686), or final fixation duration (*p* > 0.144).

### 3.3. Motor Behaviour

**Figure 4:**
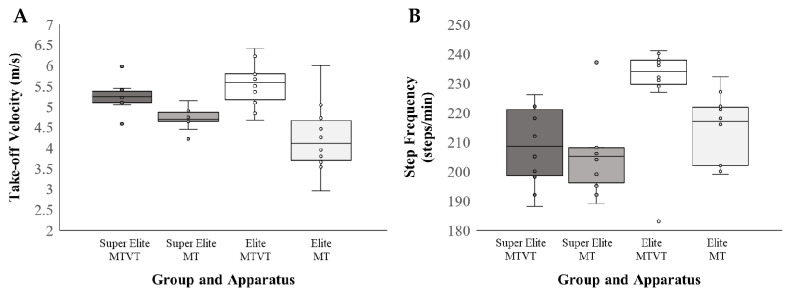
Kinematic measures by group (super elite vs. elite) and apparatus (MTVT vs. MT): (**A**): take-off velocity in meters per seconds; (**B**) step frequency in steps per minute.

The mean ± SD values for movement kinematics during the approach run and take-off are presented in Table 2. No main effect of Group was found for take-off velocity on either the MTVT (*p* = 0.175) or MT skills (*p* = 0.193), d = 0.621; however, a significant Group × Apparatus interaction was documented for take-off velocity, *β* = 0.766 (0.15–1.38), *t*(27.99) = 2.462, *p* = 0.020, *η*^2^ = 0.162 (Figure 4A). The super elite group displayed significantly greater take-off velocity (~0.5 m/s) when performing the MTVT compared to MT, *β* = 0.533 (0.15, −1.38), *t*(18) = 3.863, *p* = 0.001, *η*^2^ = 0.453, *d* = 1.72. The same but larger effect of apparatus (~1.3 m/s greater velocity for MTVT) was documented in the elite group, *β* = 1.299 (0.75, −1.85), *t*(14) = 4.815, *p* < 0.001, *η*^2^ = 0.603, *d* = 1.76.

A significant main effect of Group was documented for step frequency, *β* = 32.90 (9.35, 56.45), *t*(28) = 2.649, *p* = 0.012, *η*^2^ = 0.190, indicating that the elite group demonstrated significantly more steps/minute (mean step frequency = 221.65 steps/min ± 16.43) compared to the super elite group (mean step frequency = 206.45 steps/min ± 13.26) (Figure 4B). Although it appeared that these effects were isolated to the elite group displaying a greater number of approach-run steps on MT in particular, the Group × Apparatus interaction did not reach the threshold for significance, *β* = 11.80, *p* = 0.096. Additionally, no main effects of Group, Apparatus or their interactions were reported (all *p*’s > 0.175).

## 4. Discussion

We examined the influence of task constraints (i.e., the presence (MTVT) or absence (MT) of a vaulting table) on gaze and motor behaviours in elite, international level gymnasts of varying levels of expertise. Contrary to our hypothesis that gaze behaviours would be influenced by task constraints independently of participants’ levels of expertise, results showed that task constraints influence the gaze behaviours of elite participants to a greater degree than super elite gymnasts (Table 1). Specifically, elite gymnasts significantly reduced the amount of time fixating the mini-trampoline when performing on MTVT, while the super elite gymnasts did not differ in the time fixating the mini-trampoline in both tasks. As we hypothesized, motor behaviour was influenced by the task constraints (Table 2), with both groups significantly increasing their take-off velocity during the MTVT skill compared to MT. Furthermore, the elite participants demonstrated increased step frequency in MTVT compared to MT. Groups did not differ in performance scores in MT, but the super elite participants’ scores on MTVT were significantly higher than their elite counterparts. Finally, the two groups did not differ in gaze behaviours in MT, and no differences were documented between groups for the number of total fixations, final fixation dwell and duration during both skills, in opposition to what was hypothesized.

While super elite participants generally outperformed their elite counterparts, groups did not differ in performance scores on the MT skill, and decomposition of the interaction effect revealed that this was driven by performance in the more difficult MTVT skill. These findings suggest that simple tasks, typically mastered earlier in sport development, are not sufficient to detect expertise differences amongst elite level gymnasts.

Expertise level did not alter the preference of gymnasts to direct gaze behaviours predominantly towards the mini-trampoline (dwell time on MT = 51.05 ± 15.44% vs. 54.71 ± 11.90%). The amount of gaze time directed to the mini-trampoline was possibly driven by the need to regulate current position and therefore control distance and time-to-contact for the take-off phase [2,3,4,6,7,13]. Participants with less expertise spent significantly less time directing gaze to the mini-trampoline during the MTVT task compared to the MT skill (see Table 1). A meta-analysis [17] reported that gaze behaviour is indeed moderated by task complexity, and several published reports have corroborated the malleability of visual regulation using other related tasks [17,28,29,30,31]. The emergence of different visual behaviours depends on task characteristics and complexity, although the more-expert group demonstrated similar gaze behaviours when performing on MT and MTVT. It appears that expertise may lead to an improvement in how vision is used to process information from the environment [32,33], thereby allowing super-elite competitors to find common, efficient ways to use the visual system during similar, but different, complex motor tasks.

From a technical point of view, MTVT skills present additional constraints specifically during the first flight phase (i.e., from mini-trampoline to vaulting table) and the second flight phase (i.e., from take-off from vaulting table to landing) [21,34,35]. The elite group may have more difficulty during these phases of movement, resulting in a reduction in time directing gaze to the mini-trampoline during the approach run, in order to find their hand placement location on the vaulting table. We suggest that differences in level of expertise indicate that even elite gymnasts of significant skill-level (e.g., national team athletes) must learn how to efficiently use attention in order to focus on the right cues for the right amount of time during more complex vaults. As a result, super-elite gymnasts have the ability to direct their gaze predominantly to the mini-trampoline (as in MT task) to constantly perceive information and adjust their approach run, because they are not reliant on as much information extracted from the vaulting table. It is also possible that super-elite participants were using their peripheral vision to obtain visual information from the proximal edge of the vaulting table, while directing their focal gaze to the mini-trampoline [36]. The elite group seem to perceive visual information to adjust their approach run (with mini-trampoline still being the most fixated), as well as directing their gaze to the vaulting table to precisely place their hands (i.e., the elite group spent ~10% more time on average fixating on the vaulting table). Therefore, in applied practice, one strategy to improve MTVT skill execution would be to train hand location on the vaulting table so gymnasts can learn to relate kinaesthetic feedback with the use of limited (or peripheral) visual information from the vaulting table. Additionally, and since the mini-trampoline is the most relevant visual AOI for these tasks, we suggest the implementation of strategies such as using colours to increase attunement to this AOI. In this way, we are manipulating the task constraint to lead gymnasts to improve their gaze behaviours and motor performance.

Take-off velocity did not differ across groups, but all participants demonstrated greater take-off velocity during the MTVT (see Table 2), highlighting that motor behaviour is influenced by task constraints. As we predicted, greater velocities during the approach run and take-off have been shown to be associated with better performances and higher scores on vaulting [19,21,22,37]. The higher level of complexity on the MTVT task likely explains this discrepancy in take-off velocities between apparatuses [20,37]. Additionally, the elite group displayed significantly increased step frequency on MTVT, which has been demonstrated to be positively correlated with approach run velocity for handspring entries on the vaulting table [38]. Given the higher scores observed for the super-elite group on the MTVT compared to the elite gymnasts, in future, researchers should investigate other kinematic parameters in elite-level gymnasts that might be stronger determinants of performance outcomes on complex skills using a vaulting table.

Several limitations are worth acknowledging. First, the sample size in this study was relatively small, but gymnasts were all international level performers from the same team (Teamgym national team). We felt that securing samples of super elite and elite gymnasts would generate more insightful and impactful findings compared to using more diverse skill groupings, as per the more typical expert vs. novice design. Second, repeated trials were time consuming and quickly led to participant fatigue; therefore, we limited participants to four trials on each apparatus and took the two best trials for analysis. In future research, it would be beneficial to analyse central-peripheral awareness [36,39] and the location of the hands on the vaulting table (i.e., the distance between the edge of the vaulting table and hands), in groups with various levels of expertise. Furthermore, analysis of kinematic parameters during the approach run and contacts with the mini-trampoline and vaulting table, namely, joint angles and angular velocity, may provide detailed insights into how task constraints influence performance across various levels of expertise in gymnastics. These approaches may facilitate the design of training programs that focus on the most relevant AOIs from the environment, but essentially on its relationship with motor behaviour. In this way, gymnasts can improve their ability to select important visual information to improve motor performance.

## 5. Conclusions

We provide novel knowledge about how task constraints influence gaze and motor behaviours in elite, international level gymnasts when performing skills with similar technical structures but different levels of complexity. We also addressed how these factors change as a function of expertise. When performing skills that require a vaulting table, the amount of gaze time directed to AOIs from the environment distinguishes super elite from elite gymnasts. In contrast, the motor behaviours were less indicative of gymnast expertise level overall, and expertise level was less predictive of performance on the relatively-easier MT skill. These findings underscore the importance of visual perception for effective motor regulation and performance during vaulting skill execution in international level gymnasts.

## Figures and Tables

**Table 1:** The scores and gaze behaviour measures (mean ± SD) by group for vaulting skills executed using the mini trampoline without the vaulting table (MT) and with a vaulting table (MTVT).

	MT		MTVT	
	Super Elite	Elite	Super Elite	Elite
Score	9.64 ± 0.20	9.45 ± 0.19	9.32 ± 0.14	8.77 ± 0.37 ^a,b^
Fixations mid run (#)	1.10 ± 0.74	1.20 ± 0.79	1.50 ± 0.85	1.50 ± 0.71
Fixations end run (#)	2.00 ± 1.70	1.80 ± 0.79	1.80 ± 1.55	1.50 ± 1.08
Fixations MT (#)	3.30 ± 2.21	3.20 ± 1.48	3.10 ± 1.73	3.10 ± 1.66
Fixations VT (#)	—	—	1.40 ± 0.52	2.20 ± 1.55
Total fixations (#)	8.70 ± 2.58	7.60 ± 1.84	8.10 ± 1.91	9.00 ± 1.89
Avg. fixation duration (ms)	291.47 ± 83.85	383.21 ± 112.20 ^a^	357.78 ± 98.05	286.81 ± 96.10 ^a^
Dwell time mid run (%)	12.56 ± 11.31	14.31 ± 9.51	14.11 ± 11.86	17.05 ± 9.88
Dwell time end run (%)	22.29 ± 14.07	19.28 ± 13.38	19.36 ± 13.61	15.49 ± 7.79
Dwell time MT (%)	51.05 ± 15.44	54.71 ± 11.90 ^b^	49.46 ± 13.08 ^b^	33.08 ± 8.98 ^a,b^
Dwell time VT (%)	—	—	17.08 ± 6.86	26.80 ± 10.75
Final fixation dwell (%)	17.60 ± 11.81	21.20 ± 14.07	15.4 ± 6.64	16.4 ± 5.02
Final fixation duration (ms)	488.00 ± 429.49	822.00 ± 487.12	522.00 ± 225.77	550.00 ± 165.80

Note: # = number; MT = mini-trampoline; VT = vaulting table; ^a^ denotes a significant Group × Apparatuses interaction; ^b^ denotes a significant difference between apparatus.

**Table 2:** The movement behaviour (mean ± SD) for expert and less-expert performers across skills executed using the mini-trampoline without a vaulting table (MT) and with a vaulting table (MTVT).

Measure	MT		MTVT	
**Step Length Variables**	**Super Elite**	**Elite**	**Super Elite**	**Elite**
Step −8 to −9 (m)	1.04 ± 0.24	1.09 ± 0.31	0.85 ± 0.33	1.22 ± 0.16
Step −7 to −8 (m)	1.09 ± 0.28	1.18 ± 0.34	1.09 ± 0.54	1.22 ± 0.31
Step −6 to −7 (m)	1.09 ± 0.33	1.26 ± 0.29	1.25 ± 0.28	1.29 ± 0.24
Step −5 to −6 (m)	1.27 ± 0.21	1.29 ± 0.26	1.33 ± 0.26	1.40 ± 0.09
Step −4 to −5 (m)	1.38 ± 0.18	1.49 ± 0.35	1.39 ± 0.17	1.42 ± 0.10
Step −3 to −4 (m)	1.47 ± 0.21	1.45 ± 0.20	1.47 ± 0.12	1.51 ± 0.10
Step −2 to −3 (m)	1.47 ± 0.12	1.50 ± 0.21	1.50 ± 0.11	1.58 ± 0.13
Step −1 to −2 (m)	1.41 ± 0.14	1.37 ± 0.10	1.46 ± 0.08	1.44 ± 0.20
Take-off to step −1 (m)	3.10 ± 0.28	2.97 ± 0.22	3.22 ± 0.19	3.03 ± 0.18
Avg. step length (m)	1.28 ± 0.17	1.33 ± 0.09	1.29 ± 0.16	1.39 ± 0.07
**Step Velocity Variables**	**Super Elite**	**Elite**	**Super Elite**	**Elite**
Step −9 (m/s)	3.33 ± 0.87	4.52 ± 0.96	4.14 ± 0.88	5.09 ± 0.57
Step −8 (m/s)	4.06 ± 0.77	5.01 ± 0.91	4.80 ± 0.77	5.58 ± 0.52
Step −7 (m/s)	4.58 ± 0.65	5.42 ± 0.81	5.32 ± 0.57	5.99 ± 0.40
Step −6 (m/s)	5.10 ± 0.61	5.77 ± 0.76	5.79 ± 0.52	6.35 ± 0.34
Step −5 (m/s)	5.55 ± 0.50	6.05 ± 0.65	6.14 ± 0.40	6.57 ± 0.26
Step −4 (m/s)	5.96 ± 0.49	6.27 ± 0.55	6.77 ± 0.26	7.04 ± 0.19
Step −3 (m/s)	6.29 ± 0.45	6.49 ± 0.46	6.77 ± 0.26	7.04 ± 0.19
Step −2 (m/s)	6.64 ± 0.45	6.74 ± 0.38	7.15 ± 0.29	7.32 ± 0.21
Step −1 (m/s)	6.90 ± 0.46	6.80 ± 0.30	7.33 ± 0.22	7.37 ± 0.25
Take-off velocity (m/s)	4.71 ± 0.26 ^b^	4.24 ± 0.87 ^b^	5.24 ± 0.35 ^b^	5.53 ± 0.56 ^b^
Avg. step velocity (m/s)	5.37 ± 0.54	5.89 ± 0.63	5.99 ± 0.46	6.47 ± 0.26
Step frequency (steps/min)	204.60 ± 13.38	213.90 ±12.18 ^a^	208.30 ± 13.58	229.40 ± 16.97 ^a^

Note: Mean step length and velocity calculated from last nine steps before take-off; ^a^ denotes significant main effect of group; ^b^ denotes significant Group × Apparatus interaction.

## Data Availability

The correspondent author should be contacted for more details on data.
